# The Turbulent Network Dynamics of Microbial Evolution and the Statistical Tree of Life

**DOI:** 10.1007/s00239-015-9679-7

**Published:** 2015-04-18

**Authors:** Eugene V. Koonin

**Affiliations:** National Center for Biotechnology Information, National Library of Medicine, National Institutes of Health, Bethesda, MD 20894 USA

**Keywords:** Microbial evolution, Phylogenetic trees, Horizontal gene transfer, Muller’s ratchet, Evolvability

## Abstract

The wide spread and high rate of gene exchange and loss in the prokaryotic world translate into “network genomics”. The rates of gene gain and loss are comparable with the rate of point mutations but are substantially greater than the duplication rate. Thus, evolution of prokaryotes is primarily shaped by gene gain and loss. These processes are essential to prevent mutational meltdown of microbial populations by stopping Muller’s ratchet and appear to trigger emergence of major novel clades by opening up new ecological niches. At least some bacteria and archaea seem to have evolved dedicated devices for gene transfer. Despite the dominance of gene gain and loss, evolution of genes is intrinsically tree-like. The significant coherence between the topologies of numerous gene trees, particularly those for (nearly) universal genes, is compatible with the concept of a statistical tree of life, which forms the framework for reconstruction of the evolutionary processes in the prokaryotic world.

## Introduction

When in the late 1970s and early 1980s, Carl Woese and his colleagues constructed phylogenetic trees from 16S RNA sequence alignments, the resulting phylogenetic trees were thought to have solved the problem of microbial evolution (Woese 1987; Woese and Fox 1977; Woese et al. 1990). Indeed, all kinds of bacteria and the newly discovered domain of archaea were neatly classified in these trees, not withstanding some poorly resolved deep branches. However, this new order did not last long. As soon as the first few complete bacterial and archaeal genomes became available, comparative analysis of these sequences made it obvious that the 16S RNA tree told but a small part of the microbial evolution story (Doolittle 1999a, b). The evidence of the much greater complexity and a distinct character of microbial evolution has come from two complementary lines of observations: (i) the sequenced bacterial and archaeal genomes had dramatically different gene compositions, with only a small set of core genes being universally conserved (Koonin 2003; Perna et al. 2001); (ii) topologies of the numerous phylogenetic trees that became available for scrutiny with the advent of complete genomes were rarely fully compatible with the 16S tree, and many of these trees were highly reliable indicating that the discrepancies could not be explained away by methodological artifacts alone (Koonin et al. 2001). Over the two decades that have passed since the sequencing of the first complete bacterial genomes, findings along these lines have led to a complete reappraisal of the nature of microbial evolution. The emerging understanding is that of an incessant flux of genes through genomes, or more precisely, pangenomes of microbes. The ability to accommodate new genes and even to donate genes to other microbes is likely to be an adaptive, evolvable function. Yet, all this does not necessarily imply that the tree of life has become an obsolete concept. In this brief review, I try to integrate different aspects of the “network genomics” of microbes in an attempt to outline, even if only in wide strokes, a new coherent concept of the microbial world evolution.

## The Tree of Life is Dead: Long Live Phylogenetic Trees!

The recent paradigm shift in the study of (microbial) genome evolution is most often discussed in terms of horizontal (lateral) gene transfer (HGT). Yet, the very concept of HGT is conditioned on the existence of a vertical, tree-like evolutionary standard (often referred to as the “tree of life”) (Bapteste et al. 2005, 2009; Doolittle and Bapteste 2007). Explicitly or more often implicitly, the rRNA tree or a tree made from a concatenated alignment of several dozen (nearly) universal genes coding for components of the translation system is taken to represent such a standard (Ciccarelli et al. 2006; Woese 1987). However, these phylogenies have been quickly dubbed “trees of 1 %” that reflected, at the very best, the evolution of a miniscule fraction of genes in each organism. As Dagan and Martin point out, a model that explains 1 % of the data might be in need of replacement (Dagan and Martin 2006). Taking an even more radical view, Doolittle and colleagues have suggested that “tree thinking” in biology could be irrelevant to begin with, in particular because a tree easily can be used to depict similarity relationships between objects that have nothing to do with evolutionary relationships (Bapteste et al. 2005; Doolittle and Bapteste 2007).

Thus, the findings of microbial genomics have put into focus arguably the most basic question on evolution: is the tree of life simile touted by Darwin as the accurate depiction of the evolutionary process (Darwin 1859) a sheer illusion, at least as far as microbial evolution is concerned? I submit that this is not the case, and the “tree of life” remains a cornerstone of evolutionary biology although it has to be re-conceptualized in the light of the findings of evolutionary genomics. The argument is twofold, coming first from purely theoretical considerations and second, perhaps most important, from phylogenomic analysis. Conceptually, the history of cells is obviously a history of cell divisions and hence a tree-like process. More than that, genome replication is an inherently tree-like process as well; its tree structure is only disrupted by various forms of recombination that, however, can be quite frequent (as we discuss below). At sufficiently large evolutionary distances to eliminate homologous recombination, recombination within genes becomes deleterious and thus is rarely fixed, orders of magnitude less frequent than recombination between genes. Accordingly, gene evolution is an intrinsically tree-like process (Koonin and Wolf 2009).

**Fig. 1:**
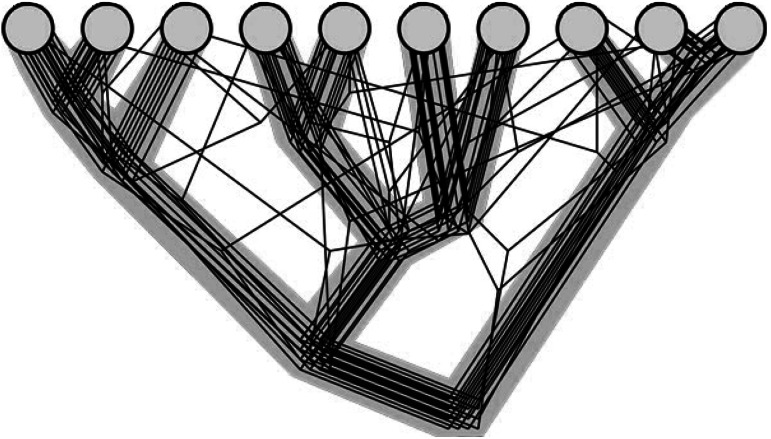
The statistical tree of life. The *gray* *background* shows the central vertical trend. The depicted “forest of life” consists of 16 trees with 20 deviations from the central trend. Reproduced from (Puigbo et al. 2013) the Creative Commons Attribution License

With respect to genome evolution, the validity of the tree simile (using Darwin’s language) remains an open question. The answer hinges on the existence of pronounced, coherent trends in the “phylogenetic forest,” i.e., the entirety of individual gene trees. More specifically, does a tree of a universal gene reflects solely the evolutionary history of that gene or does it carry information on the evolution of other genes, and if so, how many genes and how much information? In a phylogenomic study that was specifically designed to address this question, my colleagues and I performed an exhaustive comparison of the topologies of thousands of phylogenetic trees of conserved eukaryotic genes (Puigbo et al. 2009, 2014). The results clearly indicate that the trees of the (nearly) universal genes, which encode primarily the translation system components, are not only highly consistent among themselves, but also with trees of numerous other genes. In quantitative terms, the consensus topology of the nearly universal trees (the notorious tree of 1 %) accounts for almost 40 % of the variance in the tree topologies across the “forest” (Puigbo et al. 2010). Furthermore, this tree-like signal reflecting the vertical inheritance of genetic information is by far the strongest trend in the “forest of life” because the remaining variance in tree topologies reflects largely the random gene exchange. Thus, the “tree of 1 %” is not a failed hypothesis on genome evolution (Doolittle 2009) but rather a meaningful representation of the central current of genome evolution that can be legitimately construed as a “statistical tree of life” (STOL) (O’Malley and Koonin 2011) (Fig. 1). The STOL does not represent most (over 60 %) of the information flux that occurs during microbial evolution but it is the natural framework for reconstruction of these horizontal evolutionary currents.

## The Turbulent Dynamics of Microbial Evolution

A key observation of microbial genomics is that the genomes of organisms that are very closely related in terms of the sequence similarity of the universal genes (e.g., have identical 16S RNA sequences) often substantially differ in their gene repertoires (Perna et al. 2001). Thus, comparative genome analysis can be informative of both the patterns and the dynamics of genome evolution. The observations on the strong vertical evolution trend in the “forest of life” described above provide justification for the use of the tree of universal genes as a scaffold for evolutionary reconstruction (usually known as species tree). For groups of microbes at the species or genus level, such trees can be highly accurate. Given a species tree, all the genes in the pangenome of a species or an otherwise defined group of microbes (i.e., the entirety of the genes represented in the available isolates of the given group) can be mapped to the leaves of the tree. Thus, mapping then can be used to reconstruct the evolutionary scenario for the pangenome, i.e., the history of gene gains, losses, and duplications. In the early days of evolutionary genomics, such reconstructions were performed using the simple parsimony approaches that select the scenario with the minimum number of events (Kunin and Ouzounis 2003; Mirkin et al. 2003; Snel et al. 2002). Subsequently, more sophisticated maximum likelihood methods have been developed that employ evolutionary birth-and-death models to derive statistical estimates for the number of different genomic events associated with each branch of the species tree (Csuros 2010; Csuros and Miklos 2009).

A recent application of the maximum likelihood approach to the reconstruction of evolution for diverse groups of closely related bacteria (and one archaeal group) has revealed a striking picture of genomes in turmoil (Puigbo et al. 2014). Although the rates of gene gain, loss, and duplication differ by orders of magnitude across the bacterial diversity, in the most dynamic groups, several gains and losses can occur during the time that takes for the genome to accumulate, on average, one nucleotide substitution per gene. A further unexpected finding is that the most common process of genome dynamics is actually loss of genes. The estimates indicate that there are two to three times more losses than gains per nucleotide substitution (used in this case as the unit of time). Clearly, in the long term, excess of gene losses would lead to genome degradation and eventually extinction, and such is indeed the fate of many lineages, in particular those including parasites and symbionts (Merhej et al. 2013). More generally, however, the gradual gene loss seems to be off-set by bursts of gene gain that might accompany the emergence of major, phyla level and higher, groups of prokaryotes (see more below on such bursts of innovation) (Wolf and Koonin 2013). Remarkably, the rates of gene loss and gain are at least an order of magnitude greater than the gene duplication rate (Puigbo et al. 2014; Treangen and Rocha 2011).

The observations on the dynamics of microbial genome evolution clearly show that, at least in this part of the biosphere, evolution does not primarily proceed via the Darwinian route codified in the Modern Synthesis of Evolutionary Biology, i.e., by accumulation of numerous, “infinitesimally small” beneficial changes (mutations) (Darwin 1859; Dobzhansky 1937) but rather by much bigger, at least gene-sized, leaps. Furthermore, in bacteria and archaea, the dominant of genome dynamics is not “evolution by gene duplication” (Lynch and Conery 2000; Ohno 1970) that appears to be so prominent in eukaryotes, but rather, evolution by gene gain and loss.

## Pangenomes and Supergenomes of Microbes: Are There Limits to Innovation?

**Fig. 2:**
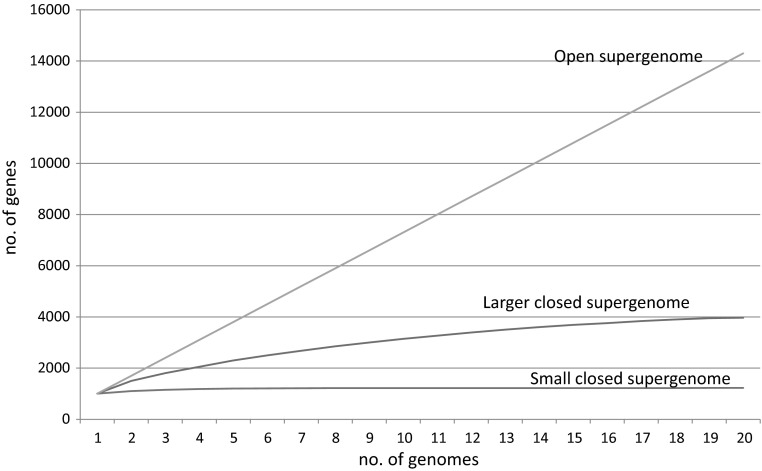
Microbial pangenomes and supergenomes. The figure schematically shows the growth of the pangenome for three types of supergenomes: small, closed (pangenome saturates after only a few genomes are sequenced); larger, closed (pangenome approaches saturation, i.e., the supergenome, as the number of genomes increases from 1 to 20); and open (no sign of saturation)

The discoveries of the frequent major differences between closely related microbes and the extensive gene gain that shapes the genomes of archaea and bacteria have changed the paradigm of microbial genomics. We now realize that the genome isolated from a bacterial colony is not a stable “blueprint” of the organism but rather a transient gene collection that, on the evolutionary timescale, can rapidly gain or lose a substantial fraction of those genes. Thus, the more relevant concepts in microbial evolutionary genomics are pangenome and supergenome (Land et al. 2015; Tettelin et al. 2005, 2008). It makes sense to differentiate between the two (Puigbo et al. 2014). The pangenome is the entirety of the genes discovered in the sequenced genomes of all isolates of a given microbial species (how to define a microbial species and even whether the notion of species makes sense for microbes, is unclear (Doolittle and Zhaxybayeva 2009); nevertheless, thousands of bacterial and archaeal genes are formally recognized, and for the sake of simplicity, I discuss pangenomes and supergenomes at the species level although in principle, both can be defined for any group of organisms). The pangenome thus is a moving target, and its size can increase with each sequenced isolate (Bosi et al. 2015). For the majority of the extensively sequenced bacterial and archaeal genomes, this is indeed the case, and signs of saturation of the number of gene are not (yet) apparent (Fig. 2). Such growing pangenomes are often called “open.” Some microbes, however, have closed pangenomes that saturate after only a few isolates are sequenced; a notable case of a closed pangenome is the (in) famous pathogen *Bacillus anthracis* (Tettelin et al. 2008).

The supergenome can be defined as the entirety of the genes that are accessible for gain to the isolates of a given species. In principle, the supergenome and the pangenome become one and the same when all isolates on earth are sequenced, i.e., the supergenome is the limit to which the pangenome tends (Fig. 2). In practice, obviously, the supergenome cannot be characterized directly, and its size has to be inferred from the available genomic data (Baumdicker et al. 2012; Bosi et al. 2015; Collins and Higgs 2012; Lobkovsky et al. 2014). Several mathematical models have been developed for this purpose. Conceivably, the simplest approach infers the supergenome size from the number of repeated gains of the same gene family detected in isolates of the same species. Intuitively, if the same genes are gained all the time, the supergenome is quite small, whereas if all gains are unique, the supergenome is operationally infinite. A maximum likelihood estimation of the supergenome size for a variety of bacteria based on this simple approach has yielded surprisingly consistent estimates of supergenomes exceeding the typical size of the genome for the given species about tenfold. In some groups, however, the supergenomes did appear “infinite” (Puigbo et al. 2014). Given the current limited sampling of the microbial world and our still crude understanding of the patterns of gene flux, these supergenome size estimates certainly should be viewed as preliminary (Lobkovsky et al. 2014). However, the estimates as low as ten genomic equivalents will soon be put to test for many groups of bacteria and archaea.

**Fig. 3:**
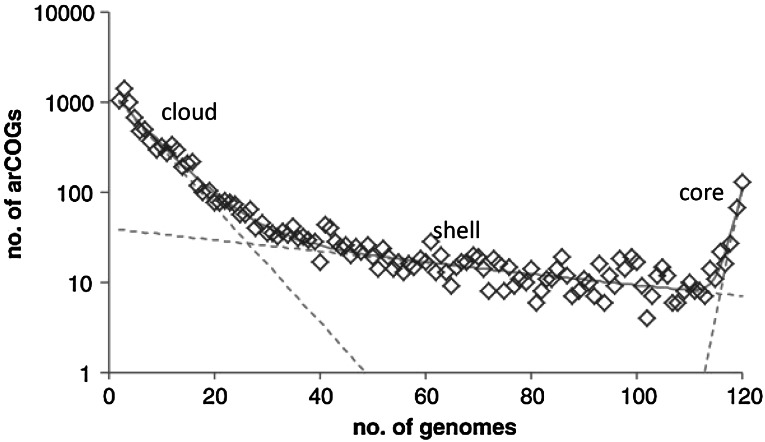
The universal distribution of gene frequencies. The plot shows gene frequencies for 120 archaeal genomes. The *dashed* *lines* show the three exponents that approximate the core, the shell, and the cloud. The *solid* *line* shows the sum of the three functions. Modified from (Wolf et al. 2012) the Creative Commons Attribution License

The gene exchange within microbial supergenomes translates into a characteristic distribution of gene frequencies in pangenomes that is remarkably well reproduced across a wide range of phylogenetic depths, from individual species to large sets of organisms representing the entire known diversity of archaea and bacteria (Koonin and Wolf 2008; Lobkovsky et al. 2013). This distribution includes three distinct components of vastly different sizes: (i) the conserved core of (nearly) universal genes that represents a small minority of the pangenomes (it is these genes, coding primarily for components of information processing systems, that give rise to the “tree of 1 %”); (ii) the moderately conserved “shell” that consists, to a large extent, of genes encoding metabolic enzymes and transport systems; and (iii) the “cloud” of rare genes that encode signaling molecules, defense systems, and a huge number of uncharacterized proteins (Fig. 3). The size of the rare gene cloud, like the size of the supergenome, is unknown but obviously, vastly exceeds the size of the shell. This tripartite distribution is an invariant in the genome universe and is, to a large extent, shaped by selectively neutral processes of gene flux. However, mathematical modeling of genome evolution shows that strictly neutral genome evolution would not produce the observed fraction of the highly conserved gene that constitute the core and much of the shell (Lobkovsky et al. 2013). The extent of the evolutionary conservation of these genes implies selection stemming from their unique functional capacities.

## The Evolutionary Impact and Adaptive Value of Horizontal Gene Transfer: Is Horizontal Gene Transfer Evolvable?

Could microbes evolve without horizontal gene transfer, simply via the competition of stable, clonal populations? Population genetic analysis indicates that such evolutionary regime is unsustainable in the long term (Takeuchi et al. 2014). Finite size clonal population typically deteriorate due to the action of the evolutionary mechanism known as Muller’s ratchet that involves accumulation of slightly deleterious mutations due to genetic drift resulting in gradual loss of fitness and eventual extinction (Charlesworth et al. 1993; Muller 1964). This appears to be the typical fate of bacteria that are confined to intracellular parasitism or symbiosis, although the action of the ratchet could be slowed down by lowering mutation rate (Allen et al. 2009). However, such mechanisms hardly can stop the ratchet altogether. It appears that the only path of escape from the Muller’s ratchet doom is gene acquisition via HGT that can result either in displacement of a mutated gene by a functional copy or by acquisition of new genes that offsets the deleterious effects of accumulating mutations (Takeuchi et al. 2014). Clearly, in prokaryotes, HGT plays the same role of preventing mutational meltdown that in eukaryotes is played by sex (Ku et al. 2015).

Escape from Muller’s ratchet could be, in a sense, the most basic role of HGT in microbial evolution but it certainly is not the only one. Acquisition of new genes and whole suits of genes, such as operons, appears to be the principal way of expanding metabolic networks in microbes (Andersson 2009; Treangen and Rocha 2011). Furthermore, as the network grows, gain of only one enzyme is increasingly likely to be beneficial, by providing access to a new nutrient (Maslov et al. 2009).

Massive gene gain via HGT appears to be the driving force behind the origin of major groups of organisms. Recent extensive search of archaeal genomes for acquired bacterial genes suggests that the emergence of most if not all major archaeal clades was associated with and conceivably caused by acquisition of hundreds or even thousands of bacterial genes (Nelson-Sathi et al. 2015). The largest influx of bacterial genes was detected in mesophilic groups such as *Halobacteria* and *Methanobacteria* and apparently led to fundamental innovation, i.e., adaptation to new lifestyles and ecological niches (Nelson-Sathi et al. 2012, 2015). The eukaryotes evolved via the same scenario, with the obvious, important distinction that the bacterial donor of the acquired genes was preserved in the form of the proto-mitochondrial endosymbiont (Ku et al. 2015).

Is HGT an evolvable capacity or in other words, an adaptive, selectable trait? Despite the wide spread and essential role of HGT in microbial evolution, this is not a trivial question because potentially HGT could be considered a neutral consequence of the presence of substantial amounts of DNA in the environment and of genetic processes such as bacteriophage infection that lead to gene transfer (transduction) (Bushman 2001). Numerous bacteria and archaea are competent for natural transformation that is mediated by specialized DNA intake pumps (Claverys et al. 2009). In principle, these pumps can be viewed as devices for utilization of environmental DNA as a source of nucleotides, with HGT being a fringe benefit. However, the recent demonstration that in some bacteria, the ingested DNA is specifically protected against degradation, thus facilitating HGT, implies that at least in part, natural competence evolved as a gene transfer machinery (Johnston et al. 2013). Bacterial conjugation (prokaryotic sex) appear to be another dedicated mechanism of gene transfer but this route involves only very closely related isolates and, similar to the eukaryotic sex, could be viewed as an evolutionary mechanism to escape from Muller’s ratchet (Ku et al. 2015).

At present, perhaps, the best showcase for dedicated vehicles of HGT appears to be the gene transfer agents (GTAs). The GTAs are defective prophages that form virus particles in which, however, they package apparently random fragments of the bacterial chromosome, rather than the phage genome (Lang et al. 2012). The GTAs then infect other bacteria or archaea, and the transferred DNA integrates into the recipient genome. In marine bacterial communities, the rate of gene transfer appears to be quite high and often involves distantly related organisms (McDaniel et al. 2010). A remarkable aspect of the GTAs is that they confer onto their carriers the ability to donate rather than acquire genetic material. Such a capacity could be adaptive in the context of utilization of “public goods” by microbial communities. The wide spread of GTAs appears to present strong evidence of evolvability of HGT.

## Concluding Remarks

The wide spread and high rate of gene exchange and loss in the prokaryotic world translate into “network genomics.” These processes are essential to prevent mutational meltdown in microbial populations (stop Muller’s ratchet) and are key contributors to innovation including origin of new clades with novel lifestyles. The contribution of gene gain and loss in microbial evolution is ostensibly greater than the contribution of point mutations. The strongest indication of the importance of massive gene transfer for the emergence of major clades comes from comparative genomics of archaea where influx of bacterial genes seems to have coincided with the origin of multiple phyla. The eukaryotes apparently evolved via a similar scenario, with the crucial distinction of the survival of the bacterial gene donor in the form of an endosymbiont. Bacteria and archaea appear to have evolved multiple dedicated devices for gene transfer.

Not withstanding the ubiquity and essentiality of gene transfer, tree-like processes are intrinsic to the processes of replication and cell division. Moreover, the substantial coherence between the topologies of numerous gene trees, particularly those for (nearly) universal genes, is compatible with the concept of a statistical tree of life, a central vertical trend in genome evolution. The statistical tree of life is a natural framework for the reconstruction of processes of gene gain and loss that shape the evolution of the prokaryotic world.
